# Preverbal infants produce more protophones with artificial objects compared to natural objects

**DOI:** 10.1038/s41598-023-36734-9

**Published:** 2023-06-20

**Authors:** Violet Gibson, Eszter Somogyi, Iris Nomikou, Derry Taylor, Beatriz López, Innocent Chitalu Mulenga, Marina Davila-Ross

**Affiliations:** 1 Psychology Department, University of Portsmouth, Portsmouth, UK; 2 Institute of Biology, University of Neuchâtel, Neuchâtel, Switzerland; 3 Chimfunshi Wildlife Orphanage Trust, Chingola, Zambia

**Keywords:** Evolution, Psychology

## Abstract

Protophones are considered to be precursors of speech. These vocalizations have been notably discussed in relation to toys and their importance for developing language skills. However, little is known about how natural objects, compared to artificial objects, may affect protophone production, an approach that could additionally help reconstruct how language evolved. In the current study, we examined protophone production in 58 infants (4–18 months) while interacting with their caregivers when using natural objects, household items, and toys. The infants were recorded in their home environment, in a rural area in Zambia. The results showed that the infants produced significantly fewer protophones when using natural objects than when using household items or toys. Importantly, this pattern was found only for the younger preverbal infants, and there was no indication in the data that the level of caregiver responsiveness differed with regard to the object type. Furthermore, the infants of the present work selected primarily the household items when exposed to both natural objects and household items. These findings suggest that natural objects are less likely to promote protophone production and, consequently, language skill development than artificial objects in preverbal infants, who seem to favor the latter, perhaps due to their features designed for specific functional purposes. Furthermore, these findings provide empirical evidence that the use of complex tools in social interactions may have helped to promote the evolution of language among hominins.

## Introduction

Protophones play a fundamental role in early language development^1^. These flexibly used speech-like vocalizations are produced spontaneously and independent of context from birth^2^. By the 3rd month, protophones become more phonologically diverse, largely on a phonatory rather than articulatory basis^3–5^ until the onset of canonical babbling by 10 months of age^1^. At around 3 months, human infants are also known to show functional flexibility in their protophone use—expressing different emotional states with the same protophone type to achieve different functions^6^. Early protophone development involves increasing phonological and semantic flexibility in vocal behavior^7^ and as such protophones are considered to be precursors of speech, associated with the emergence of first words in infants and expressive vocabulary^8^. Whereas the frequent use of protophones leads to refined language skills^9^, disruptions in their development are likely to signify language disorders^10^. Previous empirical research has given notable attention to protophone production in relation to a variety of objects designed to serve specific functional applications, such as toys^11^ and made similar conclusions on their beneficial impact on language development^11–13^.

Such research is, to our knowledge, still missing an exploration of the impact of natural objects on protophone production. It is, however, important to understand to what extent protophones and, thus, language skills are promoted in a natural environment from a developmental and an evolutionary perspective, where the parietal and extending opercular cortices that are linked with the use and manufacture of complex tools may have helped to promote the emergence of language^14,15^. Corballis^16^ argued that vocal language about man-made tools may have led, perhaps in form of pedagogy, to a number of important advancements in hominins during the Pleistocene epoch, which further promoted speech evolution as well as a dramatic surge in manufacturing more sophisticated objects. Some key linguistic abilities may, for instance, have arisen when communicating about foraging with tools^17–20^. Especially the multimodal communication where protophones and object-based gestures are co-occurring can be particularly insightful here^21^. Thus, the current study examined protophone production by comparing communication involving artificial and natural objects.

Most of what is known about object-based communication is linked to vocalizations involving toys. The type of toy or even just the presence of toys are known to influence the production of vocalizations in infants^22^. Specifically, infant vocal activity increases during interactions with traditional toys compared to electronic and feedback toys^13,23,24^, suggesting that the former may promote protophone production and benefit later language skills and outcomes. Such increased protophone production linked to specific toys has previously been explained by a distinctive attentional or arousal state of the infants^25,26^. It is, however, possible that an overall frequent occurrence of protophones is linked more to artificial objects, where toys are especially designed to engage children^27^.

Previous research has also shown that parents from WEIRD (Western, Educated, Industrialized, Rich, and Democratic) populations may take an active role in guiding infants’ attention to object features when interacting with specific toys^13,28^, which may partly explain how the infants behave towards the different objects. These interactions are associated with more protophones and object-specific labels which support the co-construction of object knowledge^29^. However, parental interference differs across cultures, for instance in Zambia children generally have a lot of freedom to explore their natural environment and objects themselves^30^. This suggests that, although not exclusively, much of infants’ object knowledge across cultures is acquired via observation and imitation^31,32^ shaped by social guidance and pre-established object functions^33^. However, the ability to attend to certain object functions and features develops later at around 12 months^29,34^, thus communication with objects is likely to vary throughout ontogeny.

The present work examined the production of protophones (vocants, squeals, and growls) with natural objects, household items and toys in infants aged 4–18 months. Our sample thus included infants both prior to and during the canonical babbling stage^1^, providing an opportunity to gain insight into the little understood development of photophone use. Consistent with findings in developmental and evolutionary psychology, we predicted that infants produce more protophones when interacting with artificial objects compared to natural objects, where the latter therefore provide less of a platform for language development and evolution^35^. In many African communities, learning through free play with little parental supervision is common and play activities often relate to practical skills^30^. In the current study, we tested this hypothesis by studying infants in their home environment in a rural area in Zambia. The home environment provided a limited availability of toys (e.g., toy car, teddy bear, ball), whereas natural (e.g. stick, leaf, rock) and household (e.g., mug, shoe, pen) objects were more common^30^.

In addition, we examined whether the infants show more social gazing, one of the first means of early communication (see^36^), for natural objects than for artificial objects. Previously, a study by Elsner and Wertz^37^ showed that social gazing differs in infants when interacting with mothers with real plants, natural objects, and artificial objects. Interestingly, the researchers found that the infants gazed predominantly at the real plants, perhaps to seek guidance before touching a potentially dangerous plant^37^. Thus, the pattern here is different from what we predicted for protophone production. Social gazing supports learning by offering important cues about the attended object^38^ which has been observed to be more prevalent when interacting with objects requiring more parental guidance^39^.

Considering the developmental changes in object reasoning we also expect natural and artificial objects to have a different effect depending on the infants’ age^29,40,41^. From the age of 8–12 months, the initial reliance on perceptual or sensory cues associated with objects sees a gradual integration of deeper structural principles, such as functional cues, affordances, and causal relationships^29,34,41^. Thus, long before infants can efficiently use tools themselves, they are able to perceptually distinguish their affordances and categorize them based on their function^42^. It is therefore likely that object functions are not obscure to the younger infants either and that older children may respond differently to artificial vs natural objects because they have had a longer time to familiarize themselves, through both observation and actual manipulation^43^ with these objects and therefore their salience is also guided by their growing knowledge. We would note here that the perceived functionality of certain objects is likely to change with the infants' age and how infants interact with those objects. For instance, grass, which seemingly has no obvious function, is used in Zambian rural communities for basket weaving, which parents consider a valuable skill for the children to acquire^30^.

## Materials and methods

### Participants and study site

The study participants were 58 infants, aged between 4 and 18 months (M_age_ = 10.95, SD = 4.67) living in rural Zambia. The sample consisted of 29 males (M_age_ = 9.93, SD = 4.64) and 29 females (M_age_ = 11.97, SD = 4.55). The infants were divided into two age groups, with 12 months as the cut-off age (4–11mths and 12–18mths, see Table 1), to reflect the verbal and motor developmental milestones occurring at this age. Infants typically begin to use first words, refine their articulatory precision, and continue to develop consonants at this age^44^. Independent steps and proto-imperative pointing also develop around this time, with more frequent co-occurrence of gestures and vocalizations^45^.

**Table 1:** Infant demographics with sex and age breakdown.

	All	Male	Female
	N = 58	N = 29	N = 29
Age (mths)			

	N = 32	N = 19	N = 13
4	1	–	1
5	8	5	3
6	6	4	2
7	4	4	–
8	3	2	1
9	3	1	2
10	3	1	2
11	4	2	2

	N = 26	N = 10	N = 16
12	5	3	2
13	1	1	–
14	2	–	2
15	5	1	4
16	4	–	4
18	9	5	4

The study site was located in Copperbelt Province, Chingola District (north of Zambia), in villages near the Chimfunshi Wildlife Orphanage (CWO). The villages housed between 15 and 50 inhabitants and were approximately 3–6 miles apart. The setting in which the recordings took place resembled a typical rural Zambian housing compound, with several houses in a close vicinity with open space areas used for housework, storage, and animal keeping. Houses were typically made of unburnt bricks and wattle and daub with thatched or iron sheet roofs. While the resources available to children could be considered limited (e.g., restricted number of store-bought toys), they had an uninterrupted access to household (e.g., cups, combs, bowls), and natural (e.g., stones, grass straws, sticks) objects, which they regularly explored and incorporated into play activities. With the majority of the housework completed outside (e.g., washing dishes, preparing meals) and the freedom to explore, the children were exposed daily to a variety of objects and to demonstrations of how these are used. The social environment of Zambian children is characterised by community level childcaring practices. While the mother is the primary caregiver, particularly in the first year, the child caring responsibilities with time are gradually redistributed among older siblings and next of kin^30^. A regular engagement of siblings and relatives can already be observed during the early stages of life, particularly as the child becomes less dependent on the mother. This collective approach to caregiving expands infants’ social network and offers frequent and diverse opportunities to acquire and practice communication skills.

### Data collection

The participant recruitment operated as part of research on vocal development across cultures which was conducted in partnership with CWO. Families were approached via the local school and opportunistic recruitment. The protocol employed an observational approach^46^ with focal sampling^47^ where each infant was focal sampled for one hour during a recording session. The recordings were taken outside of the infant’s home, where they spent most of their time and were familiar with the surroundings. While the social partner, who in most cases was the mother (mother: 45, mother & sibling(s): 9, sibling: 2, adult female: 2), received no specific instructions, many chose to situate themselves and the infant on a chair or cloth laid out on the ground. Both the infant and the mother remained in close proximity throughout the session (< 2 m). If the mother needed to temporarily withdraw during the recording a sibling or relative remained with the infant until the mother had returned.

The recordings were made by 5 male local research assistants who resided within the same compound or a local village. This minimized the potential adverse effect of an unfamiliar individual on the infant’s behavior^48^. Spontaneous unscripted behaviors of an infant were recorded for 60 min by a research assistant standing within 2–5 m of the participant using a Sony HandyCam DCR-TRV19E (Sony Electronics, Oradell, NJ, USA). The videos were collected independently by the research assistants throughout August–September and November–December 2019.

### Behavioural coding

Object signals were defined as instances when an infant used a physical object during a social interaction and the object movement was directed at the social partner. The frequency of each signal was coded with the onset set at the start of the initial movement typical for each signal type, e.g., an arm extension proceeding hitting. Signal conclusion was indicated by either object release, the beginning of a subsequent object signal, or a change in the infant behavior. The object had to be physically gripped by the infant with the social partner clearly visible. In rare instances where there was more than one potential social partner and the object signal did not rely on tactile contact (e.g., throwing at), we identified the recipient of the signal based on who the infant engaged with beforehand, their proximity to the subject, and/or direction of gaze. To avoid pseudoreplication^49^ if a signal repetition occurred within 3 s/s of the previous signal, it was considered to be part of the same series. If, however, the signal involved a different object or followed after 3 s/s of the previous signal, it was coded as a separate event. The coding was carried out using InterAct Mangold Lab Suite Version 2015 (Program Version 15.0.0.0—Arnstorf, Germany; 25 f.p.s.).

Sixteen types of object signals were coded (Touching, Hitting, Giving, Showing, Throwing down, Shaking, Pulling away, Throwing at, Pointing, Pretend giving, Pretend hitting, Pretend throwing, Offering, Touch/hit attempt, Poking, Hold to mouth). For each event of object signal we coded: (1) object type category (natural, household, toy), (2) infant vocalizations (protophones, cry/laughter, no vocalizations), (3) gazing at the social partner (yes vs no), (4) response of the social partner (yes vs no), and (5) symbolic object use (yes vs no).

#### Object types

Three categories of object types were defined: natural objects, household objects, and toys (see Fig. S1 for examples in supplementary information). Natural objects (9 items) referred to articles naturally occurring in the environment (e.g., leaves, sticks, fruits, etc.). Household objects (21 items) represented everyday use items (e.g., mugs, shoes, socks, etc.) and included a variety of items typically found around the house. Toys (5 items) represented store-bought (e.g., miniature replicas of cars, balls, books, etc.) or homemade objects (e.g., plastic bags wrapped around sticks) for children to play with. With the exception of a toy mobile phone (although with no batteries) all toys were non-feedback traditional toys.

#### Infant vocalizations

Vocalizations that overlapped with the object signal were coded into three categories: protophones, non-speech vocalizations (cry/laughter), and no vocalizations. Vocalizations referred to discrete sounds produced by the infant that overlapped with at least a portion of the object signal^50^. These overlaps included instances of both brief and prolonged vocalizations which may have started before the object signal was produced. We distinguished between protophones (indicative of emerging speech abilities) and non-speech vocalizations (vegetative/reflexive sounds)^2^. Protophones included three subcategories: vocants, squeals and growls, and non-speech included: cry and laughter^2^. Crying was closely tied to a discomfort/distress context, with high pitch and rising/falling melody^51^. Infant laughter featured voiced vowel-like bursts (e.g., panting and cackling) and unvoiced components^52^. When the object signal was not accompanied by vocalization, the event was coded as ‘no vocalization’.

#### Gazing at the social partner

Social gazing was coded as a categorical variable indicating the occurrence of gazing (yes vs no). Gazing was identified when an infant looked towards the direction of the social partner at any time during the event or 2 s/s before or after the signal^53^. This included looking at the face or other parts of the body (e.g., hands) of the social partner. This was to account for the infant’s body positioning when sitting on the ground or mother’s lap and facing away from the mother’s face.

#### Response of the social partner

The response of the social partner was coded as a categorical variable (yes vs no). This referred to any changes in the caregivers’ behavior that occurred within 2 s/s of the offset of the infant’s object signal^54^.

#### Symbolic object use

Symbolic use was coded when an infant produced a symbolic action with an object 5 s/s prior to the object signal (yes vs no). It referred to instances when an object was used as if it were another which was different from its canonical use (e.g., using a shoe as a plane, using a stick as a hair comb)^55^. For household objects and toys, we also coded if an object was used according to its canonical use (e.g., using a hairbrush to brush hair).

Cohen’s Kappa^56^ was used to evaluate the intercoder reliability. Thirty-one percent of randomly selected videos were coded by a second coder who had prior experience with behavioral coding and the software, and was naive to the hypotheses of the study. This was then compared to the coded material of the primary coder (VG). Good or very good agreement was found for all behavioral categories: type of object signal (κ = 0.91), object type (κ = 0.92), infant vocalizations (κ = 0.79), infant gazing (κ = 0.74), and response of the social partner (κ = 0.84).

### Statistical analyses

To examine the impact of age (in months) on the production of infants’ protophones and social gazing when interacting with natural or artificial objects, binomial generalized linear mixed models were computed. In the first model (Model 1) the dependent variable was the occurrence of protophones, in the second model (Model 2) the dependent variable was the occurrence of gazing. Both models included an interaction term [Age*Object Type] to examine whether the effect of object type on the production of protophones varies depending on the infants’ age. Since we had multiple data points for each infant, the infant ID was used as a random factor in both models to account for the variance between the children. The models were evaluated and met the assumptions of normality of residuals, homogeneity of variances, and absence of multicollinearity (see the supplementary information for corresponding values). Model 1 estimated the effect of two fixed effects [caregiver’s attentional state, infant’s sex] and one interaction effect [age*object type] on whether the infant produced protophones during object signals. Model 2 estimated the effect of two fixed effects [caregiver’s attentional state, infant’s sex] and one interaction effect [age*object type] on the occurrence of infant’s social gazing. All models successfully converged and were compared using the likelihood ratio test.

To examine object preference, caregivers’ level of responsiveness, and symbolic object use nonparametric tests were used as the data were not normally distributed. Percentages were used to account for interruptions that prevented object signals from being produced (e.g., an infant briefly falling asleep or nursing). To examine infants’ object preference, we used Wilcoxon signed-rank tests. Kruskal–Wallis was carried out to examine the differences in the percentage of caregivers’ responses across the three object types (natural vs household vs toy) with the epsilon-squared test used to estimate the effect size^57^. Post hoc pairwise comparisons were carried out using Dunn’s test with Holm’s corrections to control for familywise error rate^58,59^. Symbolic object use between natural and artificial (household & toys) objects was compared using Mann–Whitney U test.

Data excluded events when an infant produced either 0 or 100% for a specific measure. For instance, if an infant always produced protophones when using a household object, the data for that specific object type were removed. This approach generated in a total of 1385 events from 58 infants.

### Approvals

Data collection for this study adhered to the criteria of the Code of Ethics and Conduct, and Code of Human Research Ethics outlined by the British Psychological Society. The ethical review and approval for this work were conducted by the University of Portsmouth, Psychology Research Ethics Committee. Informed consents were obtained from the parents of all the recorded infants.

## Results

A total of 1385 object signal events were coded. The majority of object signals observed here were produced with household (N = 48 infants, 57.26% of all events) and natural (N = 29, 32.06%) objects. Toys accounted for only a small fraction of all signals and were used by only a few infants (N = 10, 10.69%). Forty percent (40.45%) of all events of object signals were accompanied by vocalizations of which 34.90% consisted of protophones with laughter/crying observed notably less often (N = 27, 5.56%). Protophones with artificial objects (household + toys) accounted for 25.85% of all object signals, compared to 7.58% for natural objects. A considerable proportion of object signals were produced with social gazing (71.19%). Gazing co-occurred most frequently when infants used natural objects (80.10%), followed by toys (75.54%) and household objects (67.42%).

### Object availability and choice

First, we examined object availability and object choice. Natural and household items were present in almost all videos (56 and 54, respectively, out of 58), with toys only occasionally observed (10 out of 58). When natural objects and either household objects and/or toys were available and within the infant’s reach, household objects & toys were selected significantly more often than natural objects (exact 2-tailed Wilcoxon signed-rank test, N = 37 infants: *Z* = 4.17, *p* < 0.001). Household objects & toys were also found to be preceded significantly more often by symbolic play than natural objects (exact 2-tailed Mann–Whitney U test, N(natural) + N(household + toys) = 29 + 51 infants: *U* = 539.00, *p* = 0.032), where 10.47% of signals with household objects and 10.14% with toys were proceeded by canonical use. We also compared the caregiver’s responsiveness across the three object types and found no significant differences (Kruskal–Wallis test, N(natural) + N(household) + N(toys) = 29 + 49 + 10 infants: *χ*^2^(2) = 3.74, *p* = 0.154).

### Protophone age comparison for natural and artificial objects.

A full model (Model 1) that predicted the occurrence of protophones (AIC: 1622.64, BIC: 1659.27) fit the data significantly better than the null model (AIC: 1647.75, BIC: 1658.22; χ2(5) = 35.12, *p* < 0.001). This full model was also significantly different to a reduced model without the interaction term (AIC: 1624.88, BIC: 1656.28) and indicated a better fit (χ2(1) = 4.25, *p* = 0.039). The full model was therefore selected. The proportion of marginal variance attributed to the fixed effects in Model 1 was R^2^ = 0.10.

**Figure 1:**
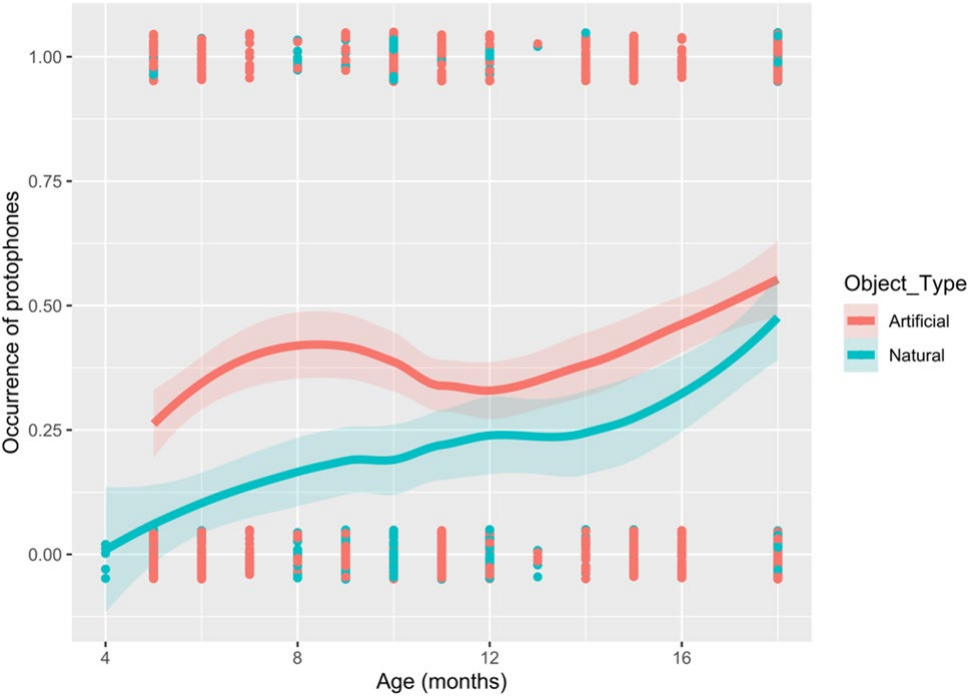
Observed occurrence of protophones by age and object type, with loess smooth and 95% confidence intervals. The red line depicts artificial objects and the blue line natural objects. The binary observations are also plotted, here jittered to reduce overplotting.

In the full model, two significant effects were found. A significant effect of object type with protophones significantly less likely to occur during interactions involving natural objects (estimate ± se =  − 1.56 ± 0.50, *p* = 0.002). There was also a significant effect of infant’s age with infants being increasingly likely to produce protophones with increasing age (estimate ± se = 0.09 ± 0.03, *p* = 0.001). There was no significant effect either of infants’ sex or caregiver’s attentional state on the production of protophones. There was, however, also a significant interaction between the infants’ age and object type (estimate ± se = 0.08 ± 0.04, *p* = 0.040) with protophones being significantly more likely to occur during interactions involving natural objects as infants’ age increased relative to the artificial objects. The observed effects of the interaction terms indicate non-overlapping confidence intervals of the curves in the first 10 months suggesting that younger infants show lower probability of protophones when interacting with natural objects compared to artificial objects (see Fig. 1). This effect appears to diminish with age, particularly around the 12 months mark where the confidence intervals between natural and artificial begin to overlap. All model values for the full Model 1 are shown in Table 2 with predicted probabilities in Fig. S1.

**Table 2:** Features of infants’ sex, caregivers’ attentional state, and infants’ age*object type as predictors of protophones (n = 58 infants).

Predictors	*B* (SE)	95% Wald confidence interval for (*B)*		Wald chi-square (*df*)	*z* value	*p* value
		Lower	Upper			
(Intercept)	− 1.64 (0.37)	− 2.34	− 0.88	19.20 (1)	− 4.30	0.000
Caregiver attending	− 0.25 (0.14)	− 0.53	0.01	3.20 (1)	− 1.87	0.073
Infants’ sex: males	0.34 (0.23)	− 0.12	0.79	2.10 (1)	1.45	0.144
Infants’ age	0.09 (0.03)	0.04	0.15	10.40 (1)	3.29	0.001
Object type: natural	− 1.56 (0.50)	− 2.70	− 0.73	9.60 (1)	− 3.41	0.002
Age*Natural objects	0.08 (0.04)	0.01	0.17	4.20 (1)	2.29	0.040

### Gazing age comparison for natural and artificial objects

A full model (Model 2) that predicted the occurrence of infant social gazing (AIC: 1439.51, BIC: 1476.14) fit the data significantly better than the null model (AIC: 1466.05, BIC: 1476.52; χ2(5) = 36.50, *p* < 0.001). This full model was also significantly different to a reduced model without the interaction term (AIC: 1442.12, BIC: 1473.52) and indicated a better fit (χ2(1) = 4.61, *p* = 0.032). The full model was therefore selected. The proportion of marginal variance attributed to the fixed effects in Model 1 was R^2^ = 0.09.

**Figure 2:**
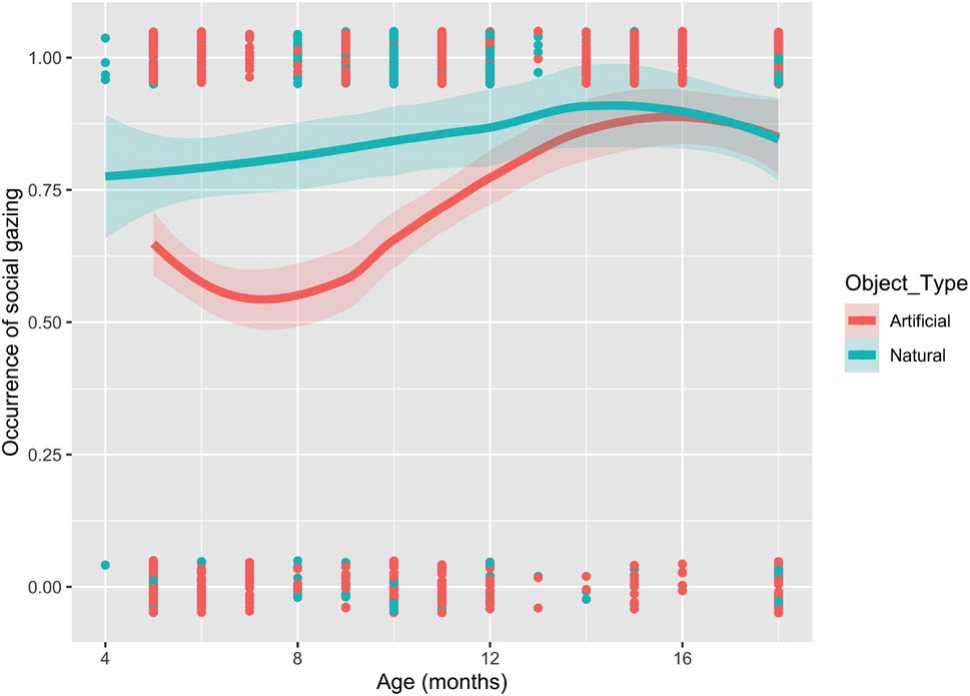
Observed occurrence of social gazing by age and object type, with loess smooth and 95% confidence intervals. The red line depicts artificial objects and the blue line natural objects. The binary observations are also plotted, here jittered to reduce overplotting.

In the full model, two significant effects were found. In this model, we found a significant effect of object type with social gazing significantly more likely to occur during interactions involving natural objects (estimate ± se = 1.50 ± 0.55, *p* = 0.001). There was also a significant effect of infant’s age with infants being increasingly likely to produce social gazing with increasing age (estimate ± se = 0.12 ± 0.03, *p* < 0.001). There was no significant effect either of infants’ sex or caregiver’s attentional state on the production of social gazing. There was, however, also a significant interaction between the infants’ age and object type (estimate ± se = -0.09 ± 0.04, *p* = 0.031) with social gazing being significantly less likely to occur during interactions involving natural objects as infants’ age increased relative to the artificial objects. The observed effects of interaction terms indicate non-overlapping confidence intervals of the curves in the first 11 months suggesting that younger infants show lower probability of protophones when interacting with natural objects compared to artificial objects (see Fig. 2). This effect appears to diminish with age, particularly around the 12 months mark where the confidence intervals between natural and artificial begin to overlap. All model values for the full Model 2 are shown in Table 3 with predicted probabilities in Fig. S2.

**Table 3:** Features of infants’ sex, caregivers’ attentional state, and infants’ age*object type as predictors of social gazing (n = 58 infants).

Predictors	*B* (SE)	95% Wald confidence interval for (*B)*		Wald chi-square (*df*)	*z* value	*p* value
		Lower	Upper			
(Intercept)	− 0.19 (0.32)	− 0.82	0.44	0.34 (1)	− 0.58	0.560
Caregiver attending	0.15 (0.14)	− 0.14	0.43	1.04 (1)	1.02	0.308
Infants’ sex: males	− 0.26 (0.20)	− 0.65	0.14	1.63 (1)	− 1.28	0.202
Infants’ age	0.12 (0.03)	0.07	0.17	21.45 (1)	4.63	0.000
Object type: natural	1.50 (0.44)	0.63	2.36	11.51 (1)	3.39	0.001
Age*Natural objects	− 0.09 (0.04)	− 0.16	− 0.01	4.68 (1)	− 2.16	0.031

## Discussion

The present study examined naturalistic caregiver-infant interactions using objects focusing on vocal and gaze behavior in Zambian infants. A special focus was hereby placed on protophones, vocalizations that have been recognized as important foundations of human speech^2^. The results revealed that infants produced protophones significantly less often when using natural objects compared to artificial objects (household items and toys) in social interactions. These findings provide empirical evidence that natural objects (e.g., sticks, leaves, bird feathers) may not promote as many speech-related vocalizations throughout ontogeny as artificial objects (e.g., mugs, shoes, pens). While previous research has shown that infants produce protophones differently depending on different toy types (e.g., traditional toys versus electronic or media-related toys)^13,23,24^, no empirical work thus far has compared the use of natural versus artificial objects from this perspective. Consequently, the pattern observed here highlights the different effects of natural versus artificial objects on early communication and potentially language development.

There was no indication in our data that the mothers' behaviors accounted for the difference in infant protophone production found in this study. Although previous studies showed that the responsiveness of mothers from WEIRD populations differs across toys^13,23,39^, the lack of such a pattern in this study could be explained by cultural differences, where in many non-Western lifestyle communities infants are not actively encouraged to lead in interactions and instead are thought to be responsive and alert to maternal expectations^60^. Perhaps a more detailed analysis of subtle behavioral changes such as changes in facial expressions using the Facial Action Coding System (FACS), that allow for changes in the visually discernible facial movements to be detected [see: ^61^], would provide further insight into this matter.

There is also a possibility that the frequent vocalisations related to artificial objects may be attributed to infants’ own object associations formed based on their past experiences^62,63^. For instance, infants may engage in particularly exciting social interactions with their older siblings that could contribute to artificial objects being subsequently associated with exciting interactions. Since such association may transfer to future interactions^62^, such as those with mothers examined in this study, the encounters with these objects may prompt more expressiveness independently of the mother’s behaviour. Yet, while infants in the present study could have produced more protophones with artificial objects because of the exciting interactions with older siblings, the features of artificial object still promoted the excitement in the initial activity.

Previous research suggests that the quantity of protophone production reflects an attention/arousal level^25,26^. We therefore argue that the infants in this study produced fewer protophones with natural objects, as they were less motivated to interact with them than with the artificial objects. In particular, toys are designed to trigger the interest in infants and children, and other artificial objects are likely to have a similar impact, perhaps due to their functional applications^27^, whereas natural objects are not designed in such way. In support of such difference in how natural objects and artificial objects are perceived, data from this study showed that the infants, who were mostly exposed to natural objects and household items in their home environment, selected more the household items or toys.

The beneficial effect of certain toys on language development has been extensively reported^11–13^. Protophones accompanying object use in infants may signify infants’ readiness to learn about the objects and enable object knowledge transfers^12^. Infants acquire knowledge about objects by learning from adults and others^33^, through observation and imitation^64^. Such learning may shape infant behaviors around object types^65^ and most likely promotes interactions with artificial objects, such as household items and toys, rather than natural objects. Consistent with this idea, our findings revealed that symbolic play of the infants was predominantly linked to artificial objects (e.g., shoes were moved in a way resembling a toy car), and the infants also copied the way artificial objects were used by others in their community. In contrast, the use of natural objects was more limited to touching and hitting other objects in this study.

Interestingly, the results also showed that it was mainly the younger infants who produced the protophones differently when interacting with objects. This could be explained by their limited ability to attend to certain object features and object knowledge being largely constructed around broader object categories^34^. In contrast, the protophone activity of older infants might relate more to object properties as opposed to object types, facilitated by their improved ability to detect and subsequently explore object features and functions. With the ontogenetic shift in object perception from form to function^41^, the perceived boundaries set by the original object categorization between natural and artificial objects are likely to diminish. Although, it is also important to consider developmental differences in the protophones themselves and their possible impact on age-related findings. For instance, by 10 months of age, most infants have reached the canonical babbling stage^1^, meaning protophones in younger infants are phonologically different to those of older infants owing to the articulatory changes that characterize this transition^3–5^. It could perhaps be the case then, that protophones in older infants were more often misclassified, or perhaps require a classification scheme of their own, thereby masking the difference observed in younger infants. Further research on the phonological continuity between protophones produced by precanonical and canonically babbling infants may shed further light in this issue.

Our findings on gazing further suggest differences in the infant behaviors when using natural objects compared to artificial objects. Specifically, the infants in this study gazed at the mothers significantly more often when using natural objects compared to the household items, particularly at a young age. Similar results were obtained by Elsner and Wertz ^37^, who found that gazing was higher for real plants compared to non-plant objects. Previous research suggests that such gazing may be explained by the infants seeking approval or information about the attended object (i.e., the natural object) from the adults^38^, suggesting the later decrease in social gaze during natural object use may be related to prior learning about natural objects that occurs during earlier months of development. Importantly, analyses of object preferences in older and younger infants revealed that both showed a preference for interacting with artificial rather than natural objects, which suggests the difference between younger and older infants gazing behavior is not explained by a difference in object interests. Instead, perhaps younger infants were simply more interested in artificial objects, while the potential interest in natural objects was less apparent, leading to increased gaze behavior towards the mother to assess the value of natural objects. By contrast, older infants may have been more aware of the value of different object types by virtue of their more extensive experience and did not therefore exhibit differences in their gazing behavior.

Before concluding, we point out a methodological limitation of this study that we believe highlights an interesting potential direction for future research. The results of this study hinge upon our classification of objects into natural and artificial. While we maintain that our distinction is valid both a priori based on pre-existing studies of perceptual categorization in infants of the age included in this study [e.g., ^66^] in tandem with our data which indicates infants do indeed make a distinction between these object categories^67^, we concede that other systems of categorization could be adopted and would indeed be interesting to explore. For example, objects could be classified on a functional rather than perceptual basis^68^. Since object classifications in development have recently been demonstrated to transition from primarily perceptual to higher-order classifications (i.e. animate vs inanimate, see Spriet, et al. ^66^]) this would be an interesting target for future research that could reveal developmental changes in communication that provide new insights into language development that were hidden in this study by virtue of the object classification system that we adopted. Alternative perspectives on the perceptual aspects of objects could also be considered, for example, classifying objects based on aspects such as colour, size, and shape rather than whether they are natural or artificial. This is particularly important since, for example, natural objects in this study were often not as vibrantly colored as artificial objects and that children show flexibility and change how they categorize objects depending on the demands of the situation^69^.

All in all, the present work reveals that natural objects used in this study are less likely to promote protophones than artificial objects in younger, preverbal infants. Protophones in association with artificial objects, on the other hand, occurred regularly in this study (accompanying on average every fourth object signal). Thus, such man-made objects most likely offer notable opportunities for preverbal infants to practice speech-related skills, meaning that their availability and their interactive use promote language development. As this impact was found in the youngest of the infants in this study, such conclusions can arguably be made in terms of universalities, although future research on this topic across human cultures is needed to support our claim, especially as infants’ encounters with objects occur in a sociocultural context that may be mediated by adults^70^. Our findings are also in line with accounts of speech evolution, where the use and manufacture of complex tools may have helped to promote language evolution within the hominin lineage^14,16,17,19^.

## Supplementary Information

Supplementary Information.

## Data Availability

The datasets used and/or analyzed during the current study are available from the corresponding author on reasonable request.
