# Supplementary material for: Preverbal infants produce more protophones with artificial objects compared to natural objects
Source: Sci Rep. 2023 Jun 20;13:9969. doi: 10.1038/s41598-023-36734-9 (PMC10282070; doi:10.1038/s41598-023-36734-9)
Supplement: Supplementary file 1 — Supplementary Information. [file 41598_2023_36734_MOESM1_ESM.pdf]

SUPPLEMENTARY INFORMATION

Preverbal infants produce more protophones with artificial objects compared to natural objects.

Violet Gibson[1*], Eszter Somogyi[1], Iris Nomikou[1], Derry Taylor[2], Beatriz López[1], Innocent Chitalu Mulenga[3], & Marina Davila-Ross[1]

[1] Psychology Department, University of Portsmouth, King Henry Building, King Henry 1st Street, Portsmouth, PO1 2DY, United Kingdom
[2] Institute of Biology, University of Neuchâtel
[3]Chimfunshi Wildlife Orphanage Trust, Chingola, Zambia

Statistical analysis

Model evaluation

Model 1. The variance inflation factor (VIF) was used to estimate collinearity between the fixed factors. As a general rule the VIF should not exceed 10 [53,54], which was the case in the full model which indicated a low to medium correlation (VIF: 1.02 – 8.81). Overdispersion measures of the model showed no significant violations ($\chi 2$ = 1303.28, p = .920). The relationship between the fitted values from the model and the simulated residuals suggested that the full model adequately captured the variation in the data. Comparing the distribution of the simulated residuals to a normal distribution showed no violations of the simulated residuals. The variance estimates of random effects was 0.38 (SD = 0.62).

Model 2. The variance inflation factor (VIF) indicated a low to medium correlation for fixed factors correlation (VIF: 1.03 – 6.84). Overdispersion measures of the model showed no significant violations (χ^2 = 1308.11, p = .910). The relationship between the fitted values from the model and the simulated residuals suggested that the full model adequately captured the variation in the data. Comparing the distribution of the simulated residuals to a normal distribution showed no violations of the simulated residuals. The variance estimates of random effects was 0.21 (SD = 0.45). Wald Chi- Square with Confidence Intervals (CI) of 95% were used to assess each parameter in the final models. Marginal R2 values were also calculated for both models [55]. No cases of missing data were reported. The analysis was carried out using RStudio v2023.03.0+386 (RStudio, Boston, MA, USA) using the '(g)lmer' function ('lme4' and 'optimix' packages).

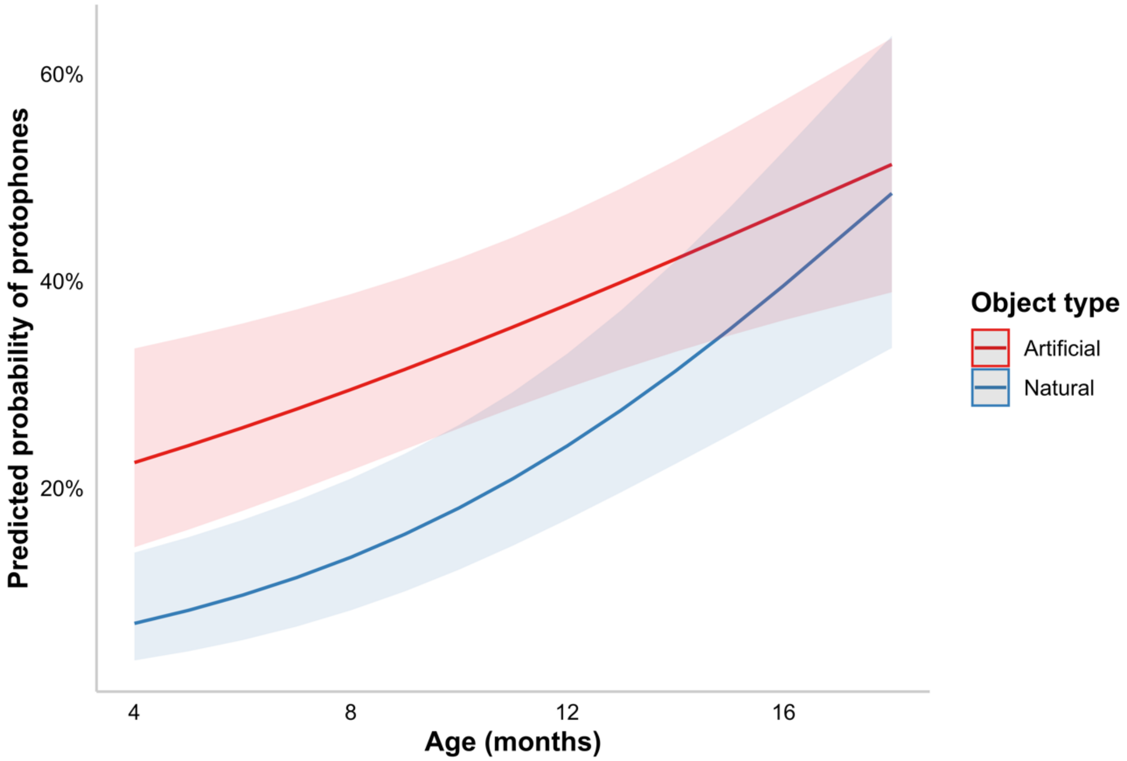

Figure S1. Predicted probability of protophones using age and object type interaction as predictors. The red line indicates artificial objects, and the blue line natural objects. The gradient shading along each line depicts 95% confidence intervals.

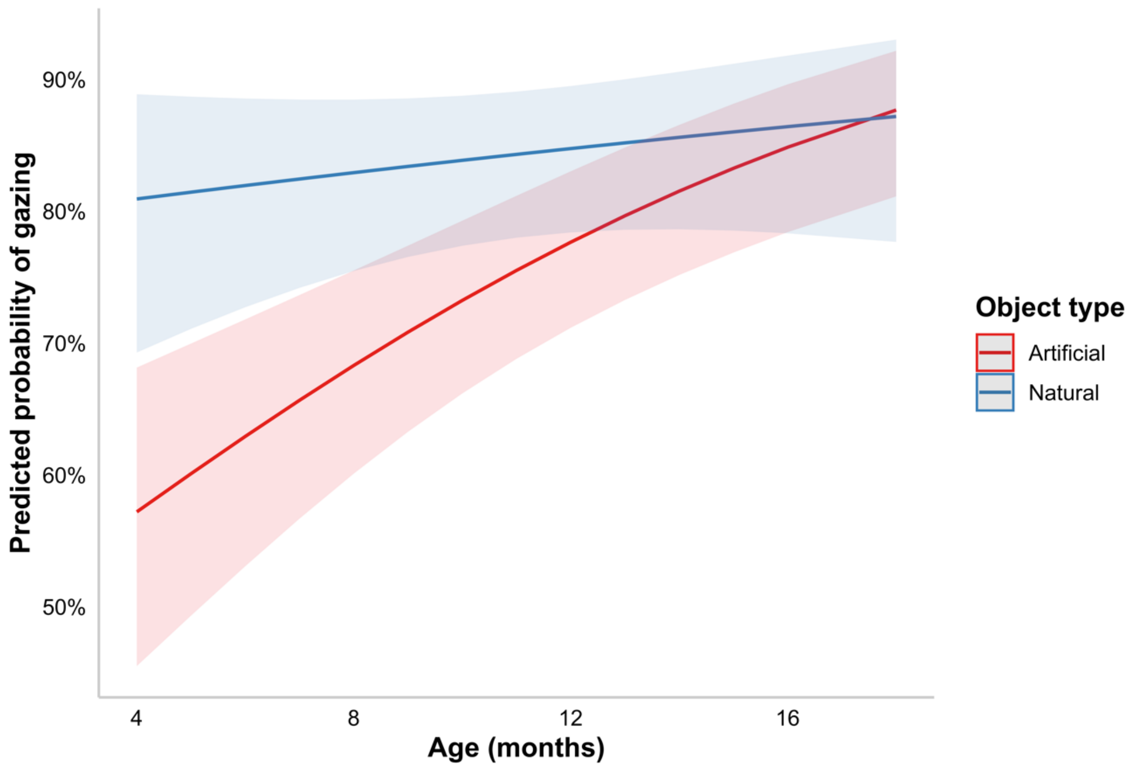

Figure S2. Predicted probability of social gazing using age and object type interaction as predictors. The red line indicates artificial objects, and the blue line natural objects. The gradient shading along each line depicts 95% confidence intervals.

Table S1. The types and varieties of objects used by the infants.

Household objects (28 types)

**Shoe**<br>(9 varieties)	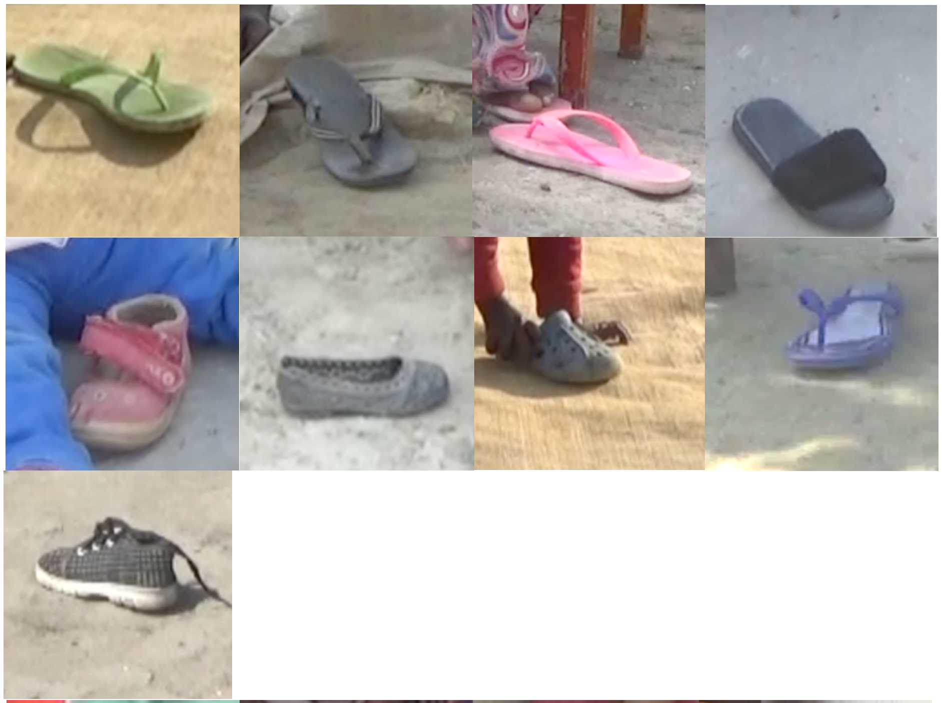
**Plastic mug**<br>(6 varieties)	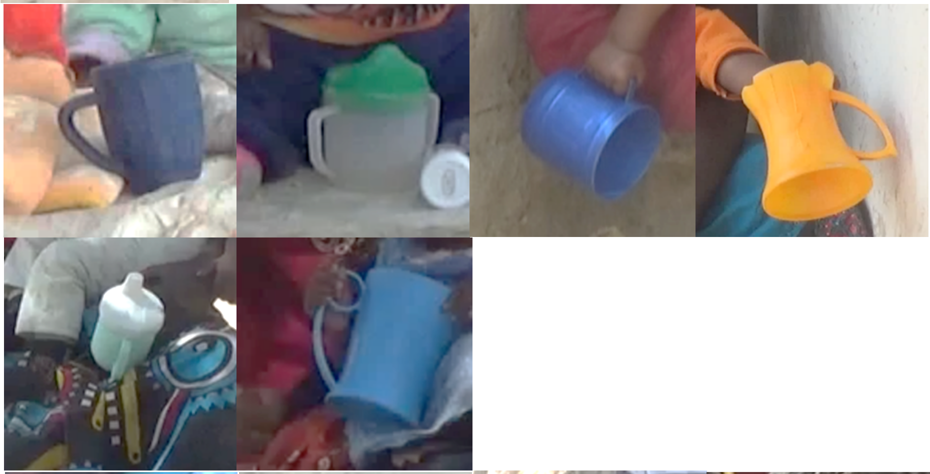
**Phone**<br>(4 varieties)	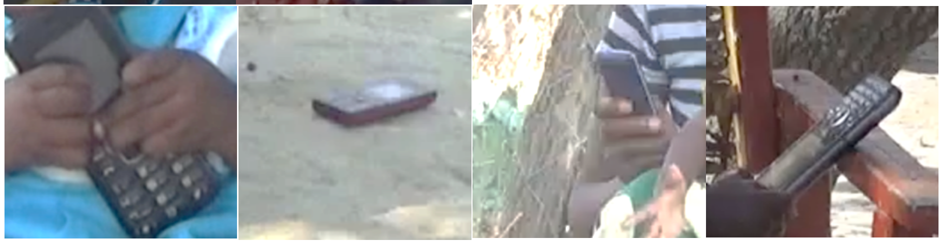

Blanket
(3 varieties)

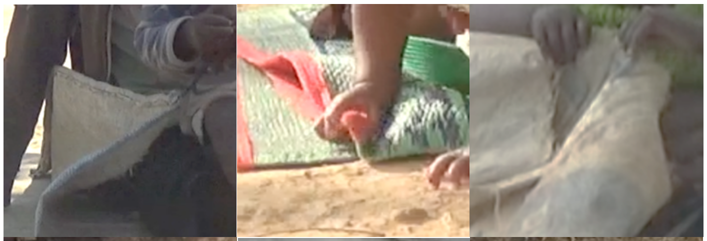

Plastic bottle
(3 varieties)

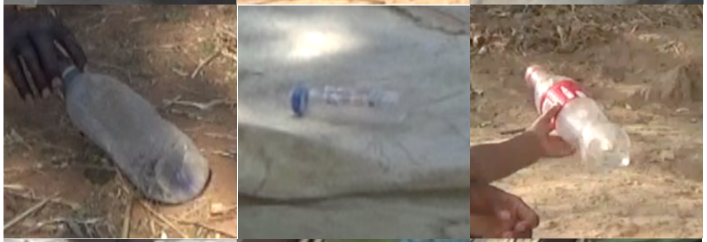

Plastic bag
(3 varieties)

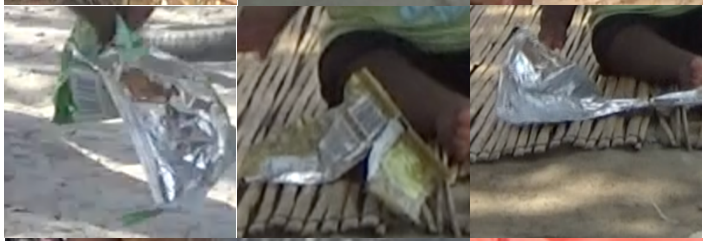

Hair band
(3 varieties)

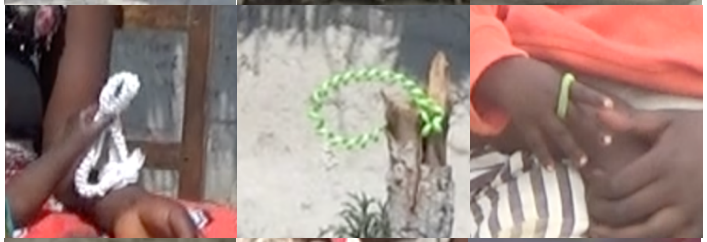

Paper
(3 varieties)

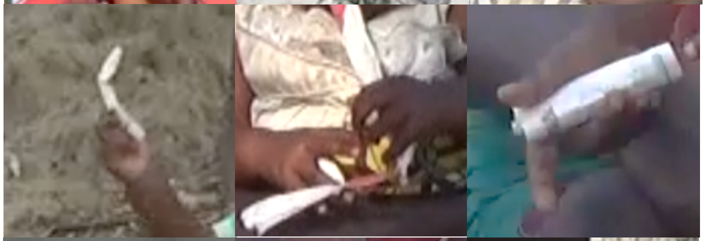

Plastic container
(3 varieties)

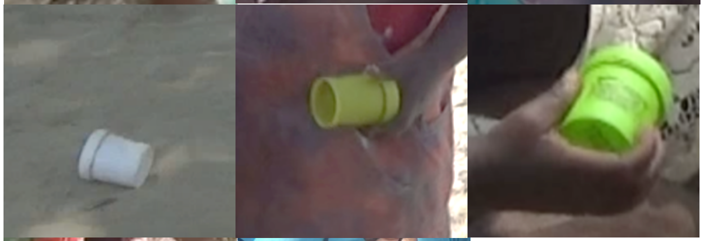

Fabric bag
(2 varieties)

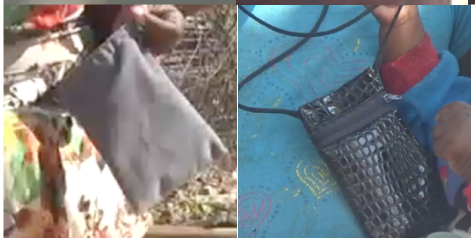

Bottle cap
(2 varieties)

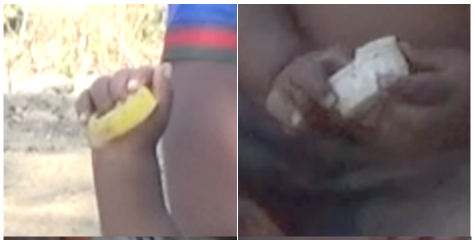

Sock
(2 varieties)

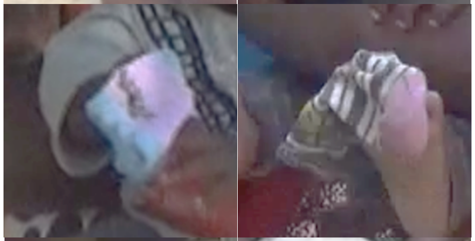

Spoon
(2 varieties)

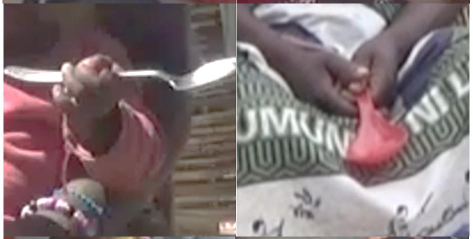

Hat
(2 varieties)

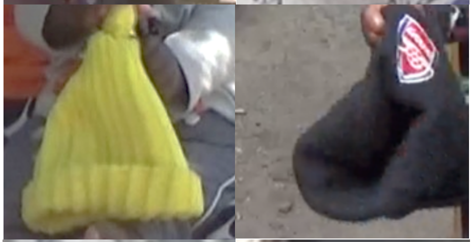

Lollypop stick
(2 varieties)

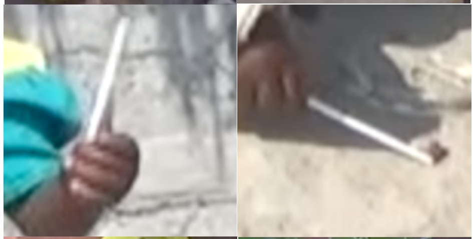

Mother's clothes
(2 varieties)

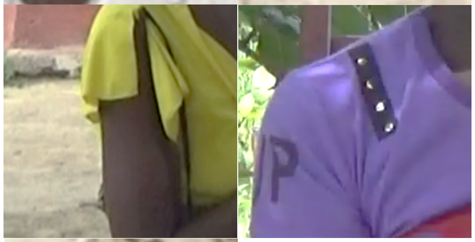

Cardboard box
(1 variety)

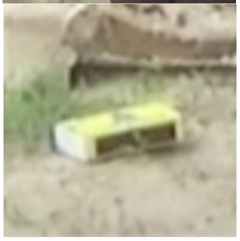

Cloth
(1 variety)

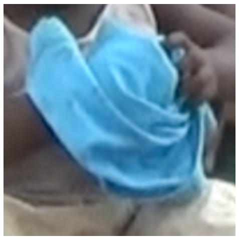

Battery
(1 variety)

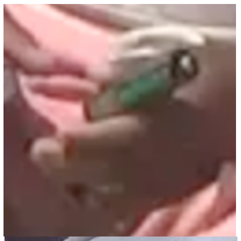

Clip
(1 variety)

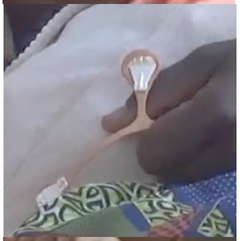

Hair comb
(1 variety)

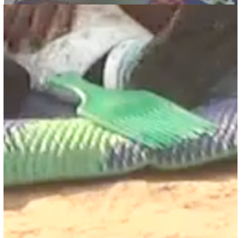

Metal rode
(1 variety)

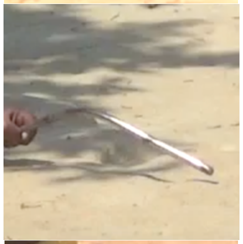

Mirror
(1 variety)

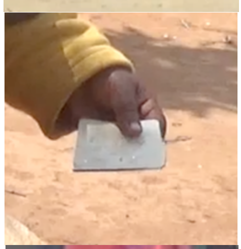

Pen
(1 variety)

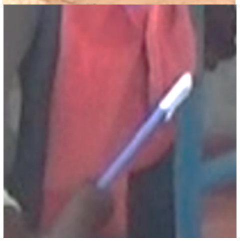

Phone Case
(1 variety)

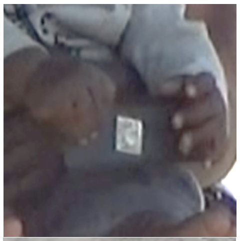

Piece of plastic
(1 variety)

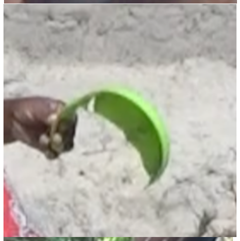

Plastic string
(1 variety)

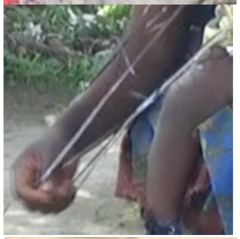

Shoe polish
(1 variety)

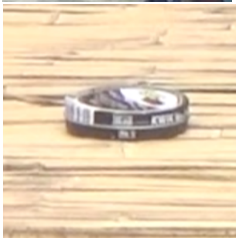

Toy objects (5 types)

Teddy
(2 varieties)

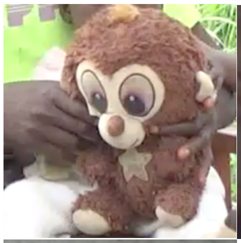
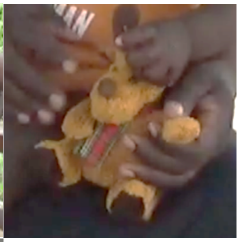

Ball
(1 variety)

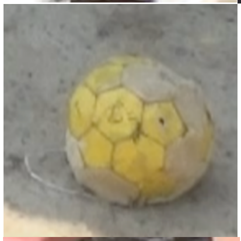

Toy phone
(1 variety)

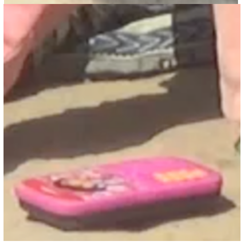

Toy trumpet
(1 variety)

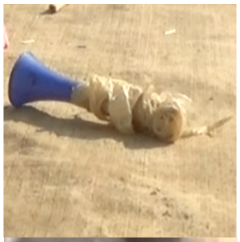

Toy wheel
(1 variety)

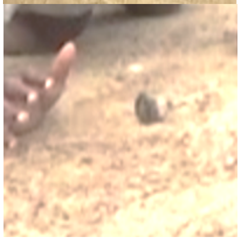

Natural objects (8 types)

Stick
(11 varieties)

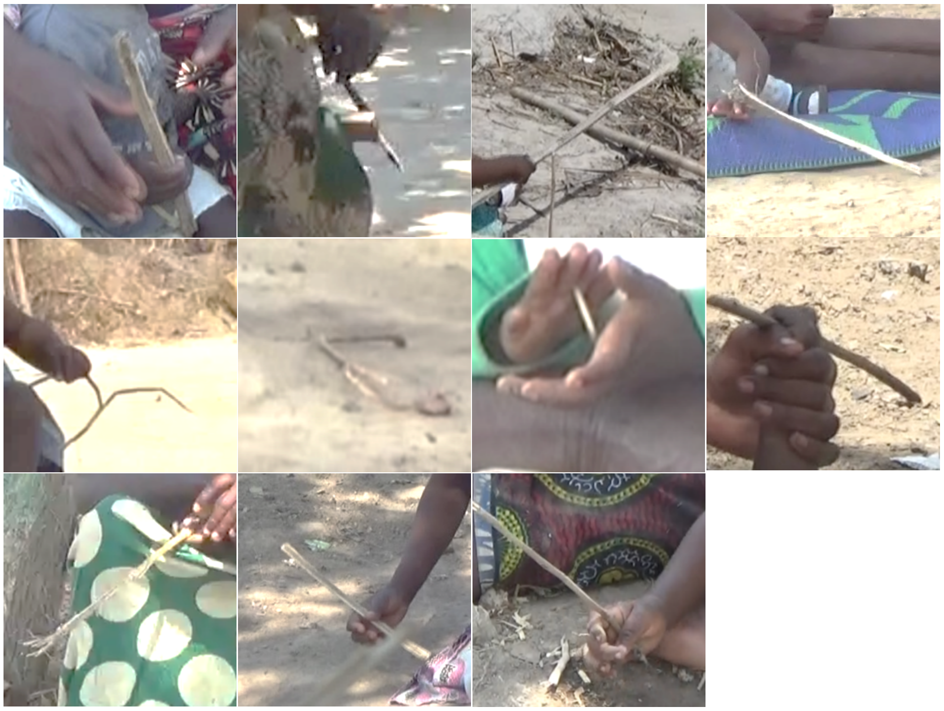

Leaf
(10 varieties)

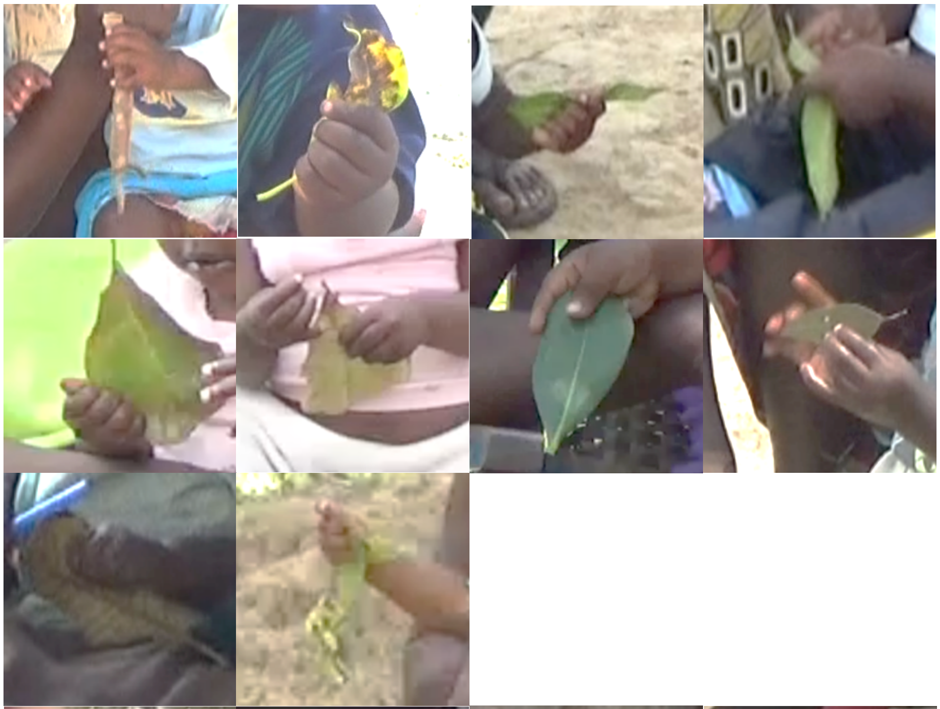

Pebble
(4 varieties)

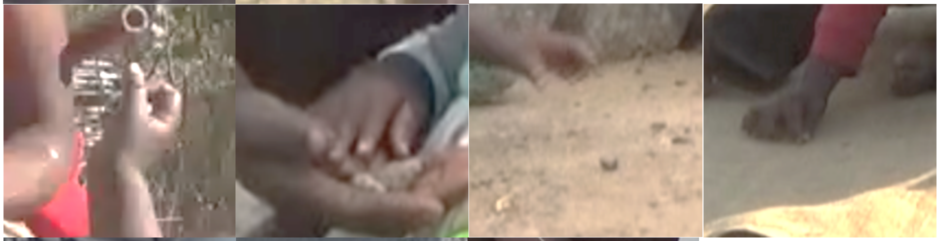

Grass/hay straw
(3 varieties)

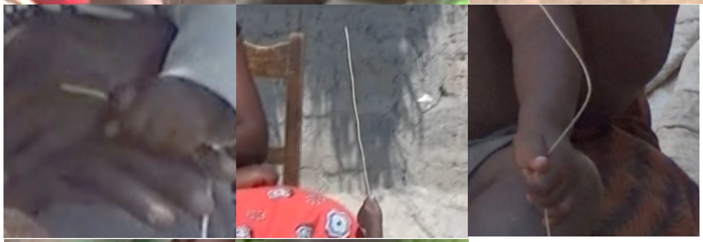

Corn cob
(2 varieties)

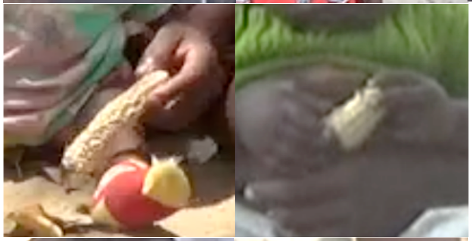

Rock
(2 varieties)

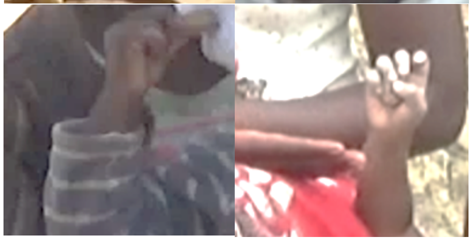

Bird's feather
(1 variety)

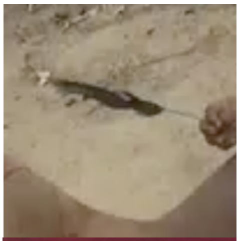

Leafy branch
(1 variety)

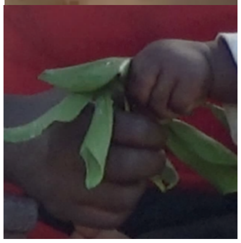

Table S2. Number of signals, protophones and gazing for 16 types of object signals. Occurrence and percentage of protophones and gazing are displayed for natural, household and toy objects (N = 58 infants).

	Frequency	Protophones			Social Gazing		
		Natural	Household	Toys	Natural	Household	Toys
	Counts	Counts | %	Counts | %	Counts | %	Counts | %	Counts | %	Counts | %
	**1385**	**102 | 23.84**	**282 | 38.86**	**80 | 42.63**	**371 | 80.96**	**581 | 67.42**	**102 | 75.54**
Touching	353	24 | 23.53	57 | 35.09	7 | 17.20	116 | 75.07	145 | 65.66	15 | 78.84
Hitting	243	10 | 10.90	38 | 33.13	16 | 45.37	60 | 81.98	95 | 68.98	14 | 82.41
Giving	200	18 | 36.31	60 | 50.18	7 | 35.48	55 | 90.81	113 | 84.97	18 | 83.33
Showing	126	10 | 36.67	41 | 45.63	4 | 100.00	26 | 92.22	67 | 73.14	2 | 50.00
Throwing down	82	4 | 14.38	10 | 21.35	10 | 58.82	12 | 55.71	14 | 43.90	12 | 70.59
Attached shaking	76	4 | 26.67	24 | 50.88	4 | 62.50	8 | 57.78	37 | 67.08	4 | 75.00
Pulling away	57	3 | 18.52	4 | 16.07	7 | 56.67	14 | 100.00	24 | 78.10	10 | 73.33
Throwing at	44	12 | 24.18	2 | 22.22	1 | 25.00	30 | 87.50	7 | 77.78	4 | 100.00
Pointing	44	5 | 68.75	15 | 74.38	11 | 95.45	8 | 87.50	9 | 51.70	2 | 54.55
Pretend giving	35	4 | 37.50	3 | 25.00	2 | 12.50	10 | 91.67	13 | 75.00	8 | 100.00
Pretend hitting	29	3 | 41.67	6 | 45.83	9 | 79.17	7 | 100.00	7 | 75.00	5 | 75.00
Offering	28	3 | 38.89	8 | 56.67	2 | 75.00	9 | 100.00	16 | 100.00	2 | 50.00
Touch/hit attempt	21	0 | 0.00	5 | 37.50	0 | 0.00	6 | 100.00	8 | 66.67	3 | 100.00
Poking	19	0 | 0.00	5 | 20.48	- | -	3 | 100.00	12 | 77.14	- | -
Hold to mouth	17	1 | 16.67	2 | 26.67	0 | 0.00	6 | 100.00	9 | 93.33	1 | 100.00
Pretend throwing	11	1 | 25.00	2 | 37.50	0 | 0.00	2 | 75.00	5 | 75.00	2 | 100.00
